# Supplementary material for: Complexome profiling of the Chlamydomonas psb28 mutant reveals TEF5 as an early PSII assembly factor
Source: Plant Cell. 2025 Mar 17;37(6):koaf055. doi: 10.1093/plcell/koaf055 (PMC12164590; doi:10.1093/plcell/koaf055)
Supplement: koaf055_Supplementary_Data [file koaf055_supplementary_data.zip › psb28_interactive.zip!/plots/intensity/qualityPlots/peptides/ERM4_Cre08.g360600.t1.1.html]

### Cre08.g360600.t1.1

Trivial name: ERM4  
SeqCoverage [median]: 3.20  
Mass: 164.88 kDa  
  
 

| Sequence | MissedCleavage | Mass [Da] | wtIntensity | muIntensity | Score |
| --- | --- | --- | --- | --- | --- |
| SAAGDSGVGGKEGGAVVAK | 1 | 1615.82 | 0.00 | 604.16 | 56.85 |
|  |  |  | 0.0 | 604.2 |  |
